# Clinicopathological Features of Superficial Non‐Ampullary Duodenal Epithelial Tumors Involving Brunner's Glands

**DOI:** 10.1002/deo2.70284

**Published:** 2026-01-26

**Authors:** Kazuki Takayama, Taro Iwatsubo, Mitsuaki Ishida, Shun Sasaki, Akitoshi Hakoda, Noriaki Sugawara, Kazuhiro Ota, Toshihisa Takeuchi, Nao Kawaguchi, Atsushi Tomioka, Ryo Tanaka, Mitsuhiro Asakuma, Sang‐Woong Lee, Kazuhide Higuchi, Yoshinobu Hirose, Hiroki Nishikawa

**Affiliations:** ^1^ Department of Internal Medicine Osaka Medical and Pharmaceutical University Takatsuki Japan; ^2^ Endoscopy Center Osaka Medical and Pharmaceutical University Hospital Takatsuki Japan; ^3^ Department of Pathology Osaka Medical and Pharmaceutical University Takatsuki Japan; ^4^ Division of Pathology Osaka Medical and Pharmaceutical University Hospital Takatsuki Japan; ^5^ Department of General and Gastroenterological Surgery Osaka Medical and Pharmaceutical University Takatsuki Japan

**Keywords:** Brunner's glands, cold snare polypectomy, endoscopic resection, submucosal involvement, superficial non‐ampullary duodenal epithelial tumors

## Abstract

**Background and Aims:**

Although Brunner's glands (BGs), located in the submucosa of the duodenum, may be involved by tumor cells of superficial non‐ampullary duodenal epithelial tumors (SNADETs), the detailed incidence and histopathological features have not yet been analyzed. This study aimed to clarify the histopathological relationship between SNADETs and BGs.

**Methods:**

We retrospectively analyzed SNADETs that were resected at a single center between 2006 and 2021. Resected specimens were histologically evaluated to determine the presence and/or involvement of SNADET in BGs. The relationship between clinicopathological features and tumor involvement in BGs was also assessed.

**Results:**

In total, 114 lesions were included. Direct connection with BGs was seen in 52.6% of SNADETs, and submucosal BG involvement was observed in 7.0% (95% confidence interval 3.1%–13.4%) of SNADETs. The presence of submucosal BG involvement was significantly associated with 0‐IIc morphology, high‐grade dysplasia or carcinoma, and the gastric mucinous phenotype. The presence of either a lesion size 10 mm or greater or 0‐IIc morphology demonstrated a sensitivity of 100%, a specificity of 34.9%, and a false negative rate of 0% for predicting submucosal BG involvement. Moreover, non‐neoplastic BGs were exposed at the vertical margin in 15.1% of endoscopic resection specimens.

**Conclusion:**

SNADETs can have submucosal involvement via BGs, particularly in lesions fulfilling either a size ≥10 mm or a 0‐IIc morphology. These pathological findings suggest that vertical resectability may be relevant in their endoscopic management, although further studies are needed to clarify clinical implications.

**Abbreviations**
BG: Brunner's gland
CI: confidence interval
CSP: cold snare polypectomy
EMR: endoscopic mucosal resection
ESD: endoscopic submucosal dissection
HE: hematoxylin and eosin
LECS: laparoscopic and endoscopic cooperative surgery
SD: standard deviation
SMT: submucosal tumor
SNADET: superficial non‐ampullary duodenal epithelial tumor
UEMR: underwater endoscopic mucosal resection

## 1 Introduction

Small bowel tumors are relatively rare, with duodenum tumors accounting for 72%; moreover, 93% are diagnosed at an early stage, resulting in a favorable prognosis [1]. A recent report from Japan indicates that the incidence of non‐papillary duodenal cancer is 23.7 per 100,000 person‐years, which is higher than the 2.9–4.3 per 100,000 person‐years reported in Western countries [2]. This is likely due to the recent spread of endoscopy as well as increased clinical attention to duodenal tumors, leading to more opportunities for diagnosing duodenal cancers in Japan [2, 3].

Superficial non‐ampullary duodenal epithelial tumors (SNADETs) are defined as mucosal or submucosal tumors originating from the non‐ampullary region of the duodenum and consist of neoplastic glandular epithelium. These are indicated for endoscopic resection [4] with various treatment methods, including cold snare polypectomy (CSP) [5, 6, 7, 8], endoscopic mucosal resection (EMR) [9], underwater EMR (UEMR) [10], and endoscopic submucosal dissection (ESD) [11, 12]. However, the optimal strategy has not been fully established, partly because the unique anatomical and histological features of the duodenum differ from those of the colon. Brunner's glands (BGs), which are located predominantly within the submucosa of the proximal duodenum, represent one such characteristic structure. The BGs are mucous glands located in the submucosal layer that open into the crypts of the duodenal epithelium. BGs have been reported to develop into SNADETs with a gastric mucinous phenotype via gastric metaplasia due to inflammation [13, 14, 15]. Since SNADETs adjacent to BGs are often observed in histopathological specimens, their spatial relationship suggests the tumor may involve BGs.

A better understanding of the histopathological relationship between SNADETs and BGs may provide important biological insight and could be informative when considering endoscopic management in the future, particularly in relation to resection depth and specimen assessment. Therefore, in this study, we aimed to elucidate the clinicopathological characteristics of BG involvement in SNADETs using resected specimens, with particular focus on the presence of tumor involvement via BGs.

## 2 Materials and Methods

### 2.1 Patients and Study Design

This study included 116 consecutive patients with 117 SNADETs (one patient having two lesions) who underwent endoscopic or surgical resection at our institute between January 2006 and December 2021. The exclusion criteria were as follows: (i) tumors involving the ampulla; (ii) familial adenomatous polyposis syndrome; (iii) specimens without a sufficient submucosal resection by CSP, cold forceps polypectomy, or hot biopsy; and (iv) lesions with positive vertical margins in resected specimens. The resection method was determined at the discretion of the physician, endoscopist, or surgeon and included: EMR, either the conventional technique with submucosal injection or the underwater technique; laparoscopic and endoscopic cooperative surgery (LECS) with serosal suturing after ESD; and surgical resection alone. CSP was not principally performed at our institute. Specimens were obtained via endoscopic or surgical resection, fixed in formalin, and embedded in paraffin.

The study protocol was approved by the Institutional Review Board of Osaka Medical and Pharmaceutical University Hospital (Nos. 2020–124 and 2024–084) and conducted in accordance with the principles of the Declaration of Helsinki. Informed consent was obtained using the opt‐out method detailed on the website of our institute.

### 2.2 Clinical Characteristics of SNADETs

Clinical data, including patient age, sex, and lesion characteristics, were obtained from medical records. Lesion size was determined from the preoperative endoscopic record, and morphology was classified into the elevated type (0‐Ip, 0‐Is, and 0‐IIa), flat type (0‐IIb), depressed type (0‐IIc), and submucosal tumor (SMT) like type. The location was classified as the bulb, second portion (pre‐ampulla), second portion (post‐ampulla), or third portion.

### 2.3 Histological Evaluation

The specimens from the endoscopic resection were sectioned at 2‐mm intervals or cut in the middle of the lesion for diminutive lesions, whereas the specimens from surgical resection were sectioned at 5‐mm intervals. At least three researchers (Kazuki Takayama, Taro Iwatsubo, and Mitsuaki Ishida), including the pathologist, performed the histopathological evaluations. SNADET histological differentiation was graded according to the revised Vienna classification and the recent World Health Organization Classification [16]. The three mucinous phenotypes were determined using immunohistochemical staining for MUC2, MUC5AC, MUC6, and CD10 for classification as follows: gastric type (expression of MUC5AC or MUC6), intestinal type (expression of MUC2 or CD10), and mixed type [expression of both (MUC5AC or MUC6) and (MUC2 or CD10)]. The immunohistochemical analyses were performed on all specimens.

**FIGURE 1:**
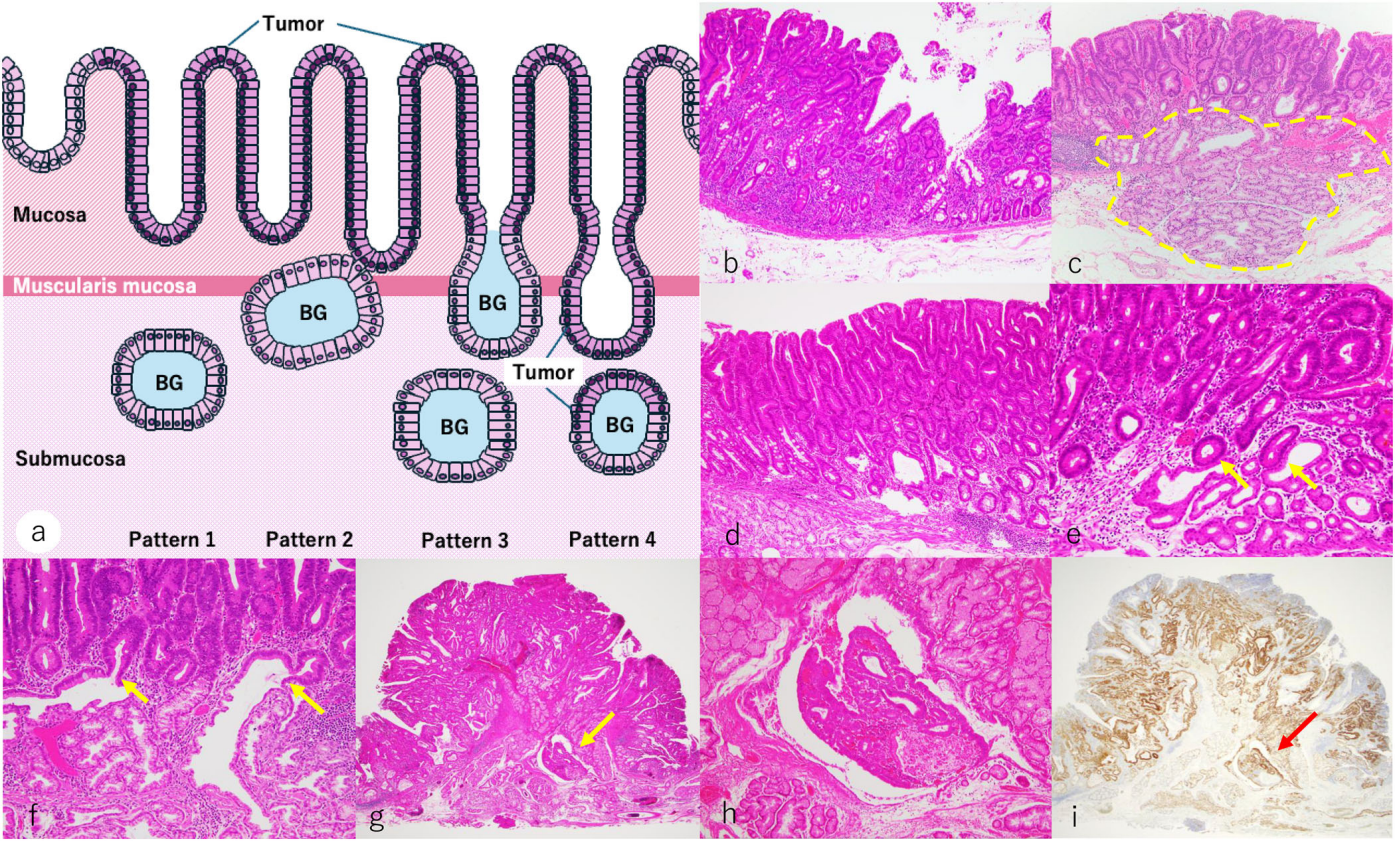
Histopathological evaluation of Brunner's glands (BGs). (a) Schema of superficial non‐ampullary duodenal epithelial tumors (SNADETs) and BGs. (b) The absence of BGs deep to SNADETs. (c) The presence of BGs (yellow dotted line) deep to SNADETs. (d) There are no connections between the tumor and BGs (pattern 1). (e) Tumor glands adjacent to BGs (yellow arrows), but neither a direct connection nor tumor front formation was present (pattern 2). (f) Involvement of the tumor into BGs in the mucosa (pattern 3). Tumor front formation in BGs is confined to the mucosa (yellow arrows). (g) Involvement of the tumor into BGs (yellow arrow) in the submucosa (pattern 4). (h) High power view of BG involvement in (g). (i) BGs are positive for MUC6. The red arrow shows involvement of the tumor within BG in the submucosal layer (pattern 4).

**FIGURE 2:**
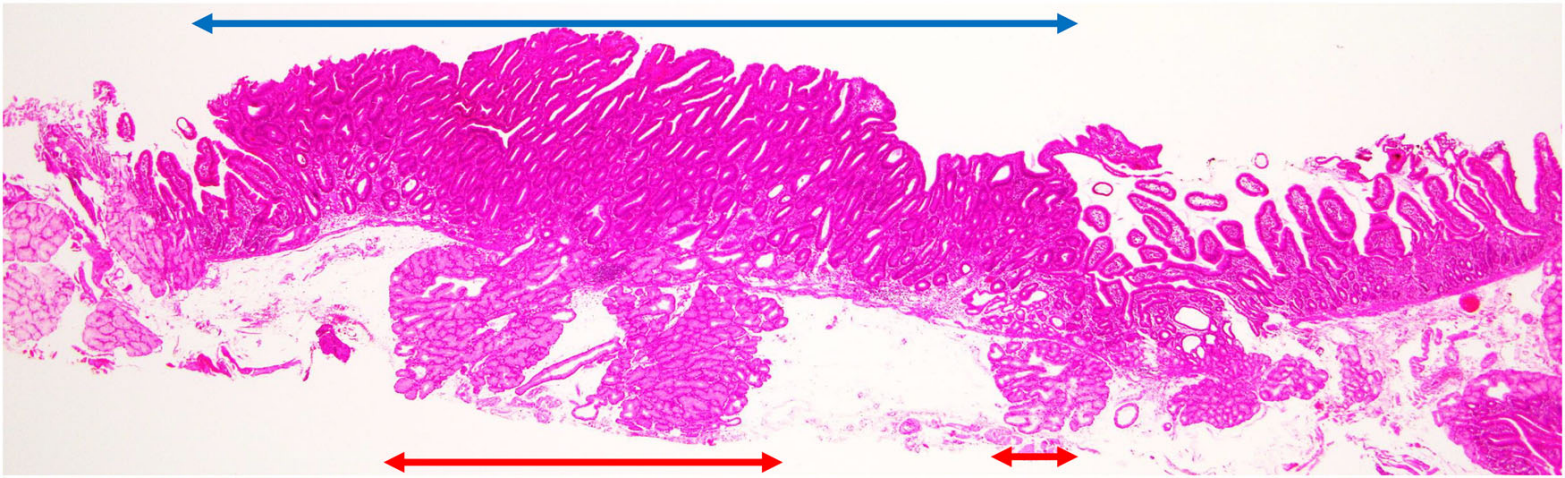
The ratio of the Brunner's glands to superficial non‐ampullary duodenal epithelial tumors (SNADETs) in the horizontal direction. It is calculated by the total distance of the Brunner's glands (red arrows) directly deep to the tumor relative to the distance of the tumor (blue arrow), which is classified into five categories (0%, 1–25%, 26–50%, 51–75%, and >76%).

BGs were identified based on hematoxylin and eosin (HE)‐stained sections as well as the expression of MUC6. The following features of BGs were evaluated (Figure 1a):

- Presence of BGs beneath the SNADETs (Figure 1b,c).
- The connection between BGs and tumor glands. The involvement of tumor glands in the BGs was classified according to the following criteria:
  Pattern 1: No connection between tumor glands and BGs, and separated from each other (Figure 1d).
  Pattern 2: Tumor glands adjacent to BGs, but neither a direct connection nor tumor front formation (Figure 1e).
  Pattern 3: Presence of a direct connection and tumor front formation between the tumor glands and BGs, confined to the mucosal layer or muscularis mucosae (Figures 1f).
  Pattern 4: Presence of a direct connection and tumor front formation between the tumor glands and BGs, extending into the submucosal layer (Figure 1g–i). This tumor expansion is confined within the existing basement membrane of BG, and they have not infiltrated beyond the basement membrane into the stroma.
- The ratio of total length of the SNADETs in the horizontal direction to that of BGs was calculated (Figure 2) and was categorized as 0%, 1%–25%, 26%–50%, 51%–75%, and more than 76%.
- Some BGs show BG hyperplasia. Presence of BG hyperplasia deep to the SNADETs, judging from the comparison of BGs deep to the tumor with those surrounding regions of the tumor (Figure S1); this is because there are no definite diagnostic criteria for the pathological diagnosis of commonly used BG hyperplasia.
- Vertical margins of non‐neoplastic BGs (but not tumor glands) in specimens that underwent endoscopic resection. If exposed ruptured BGs were present in the vertical margin, the vertical non‐neoplastic BG margin was considered positive.

### 2.4 Follow‐Up After Resection for SNADETs

Local recurrence was evaluated in patients who underwent endoscopic resection for SNADETs and were followed with endoscopic surveillance for at least 1 month after treatment, which was defined as a tumor confirmed endoscopically and histologically at the previous resection site. Surgical patients were excluded from the analysis of long‐term outcomes.

### 2.5 Statistical Analysis

Continuous variables are presented as mean and standard deviation (SD) or median and range, as appropriate for the data type. Fisher's exact tests were used to analyze categorical variables. Univariate logistic regression analysis was performed to identify the predictive factors influencing the involvement of tumors in the submucosa via BGs (pattern 4). All analyses were performed using the statistical program R, version 4.0.4 (R Foundation for Statistical Computing, Vienna, Austria), and *p* < 0.05 was considered statistically significant.

## 3 Results

### 3.1 Patients’ Characteristics and Histopathological Features of SNADETs and BGs

A total of 114 lesions from 113 patients were analyzed after excluding two lesions resected by CSP and one lesion resected by hot biopsy. Patient characteristics and endoscopic features are shown in Table 1. The mean patient age was 65.9 years (range: 31–86 years), and 70.2% were male. The mean lesion size was 12.4 mm (range: 3–40 mm), and the majority of lesions were located in the second portion of the duodenum, either pre‐ampullary (37.7%) or  post‐ampullary (43.9%). Morphologically, the most frequent type was 0‐IIa (73.7%), and one lesion showed SMT‐like protrusion. EMR was most frequently performed (71.9%), followed by ESD or LECS (14.9%) and surgical resection (13.2%).

**TABLE 1:** Clinical and histopathological features of the cohort.

	All lesions (*n* = 114)
**Sex, *n* (%)**	
Male	80 (70.2)
Female	34 (29.8)
**Age, years**	
Mean (SD)	65.9 (12.4)
Median (range)	68 (31–86)
**Lesion size, mm**	
Mean (SD)	12.4 (7.3)
Median (range)	11 (3–40)
**Location, *n* (%)**	
1st	17 (14.9)
2nd (pre‐ampulla)	43 (37.7)
2nd (post‐ampulla)	50 (43.9)
3rd	4 (3.5)
**Morphology, *n* (%)**	
0‐I	15 (13.2)
0‐IIa	84 (73.7)
0‐IIc	14 (12.3)
SMT‐like	1 (0.9)
**Treatment, *n* (%)**	
EMR	82 (71.9)
ESD/LECS	17 (14.9)
Surgery	15 (13.2)
**The presence of BGs, *n* (%)**	105 (92.1)
**BG involvement patterns, *n* (%)**	
Pattern 1	31 (27.2)
Pattern 2	23 (20.2)
Pattern 3	52 (45.6)
Pattern 4	8 (7.0)
**Connection between BGs and tumor glands**	60 (52.6)
**The ratio of BG/SNADET, *n* (%)**	
0%	18 (15.8)
1‐25%	26 (22.8)
26‐50%	16 (14.0)
51‐75%	9 (7.9)
>76%	45 (39.5)
**BG hyperplasia, *n* (%)**	62 (54.4)
**Histological type, *n* (%)**	
Category 3, low‐grade dysplasia	83 (72.8)
Category 4, high‐grade dysplasia or mucosal carcinoma	30 (26.3)
Category 5, submucosal carcinoma	1 (0.9)
**Mucinous phenotype, *n* (%)**	
gastric	9 (7.9)
intestinal	86 (75.4)
mixed	19 (16.7)

Abbreviations: BG, Brunner's gland; EMR, endoscopic mucosal resection; ESD, endoscopic submucosal dissection; LECS, laparoscopic endoscopic cooperative surgery; SD, standard deviation; SMT, submucosal tumor; SNADET, superficial non‐ampullary duodenal epithelial tumor.

Table 1 summarizes the histopathological features of the study cohort. According to the revised Vienna classification, 83 (72.8%), 30 (26.3%), and one (0.9%) lesions were classified into categories 3, 4, and 5, respectively.

BGs were identified in 105 lesions (92.1%) deep to SNADETs. A histological connection between tumor glands and BGs was observed in 60 (52.6%) SNADETs (patterns 3 and 4); 52 lesions (45.6%) were pattern 3, and eight lesions (7.0%) were classified as pattern 4. No lesions confined to the muscularis mucosae were observed among the pattern 3 lesions: all pattern 3 lesions were confined to the lamina propria. BG hyperplasia was observed in 62 lesions (54.4%). The ratio of the total length of BGs to that of SNADETs in the horizontal direction of over 76% was observed in 45 lesions (39.5%). Mucinous phenotype showed nine lesions (7.9%) of the gastric type, 86 lesions (75.4%) of the intestinal type, and 19 lesions (16.7%) of the mixed type. Only 1 lesion was classified as category 5 according to the Vienna classification; its mucinous phenotype was the gastric type, and BG involvement corresponded to pattern 2 (Figure S2).

### 3.2 Endoscopic Resection

A total of 99 lesions were endoscopically resected (82 lesions by EMR and 17 lesions by ESD or LECS). Lesions resected with ESD or LECS were significantly larger than those with EMR (22.4 ± 9.5 mm vs. 10.9 ± 5.5 mm, *p* < 0.001) (Table S1). The rate of en bloc resection was similar between the resection techniques, 69 lesions (84.1 %) with EMR and 16 lesions (94.1%) with ESD or LECS (*p* = 0.682). A total of 15 lesions (15.1%) had non‐neoplastic BGs in the vertical resection margin, which were observed in 17.1% of lesions resected by EMR and 5.9% of those resected with ESD or LECS. Among the 99 patients who underwent endoscopic resection, 80 were successfully followed up with endoscopic surveillance for at least 1 month, with a median follow‐up period of 26.5 months (range 1–123 months). During the follow‐up period, local recurrence was observed in four patients (5.0%), and in three of these patients, the horizontal resection margin was positive or unclear (Table S2). All the recurrent lesions were completely resected using subsequent endoscopic procedures.

### 3.3 The Relationship Between BG Involvement Patterns and the Clinicopathological Features of SNADETs

**FIGURE 3:**
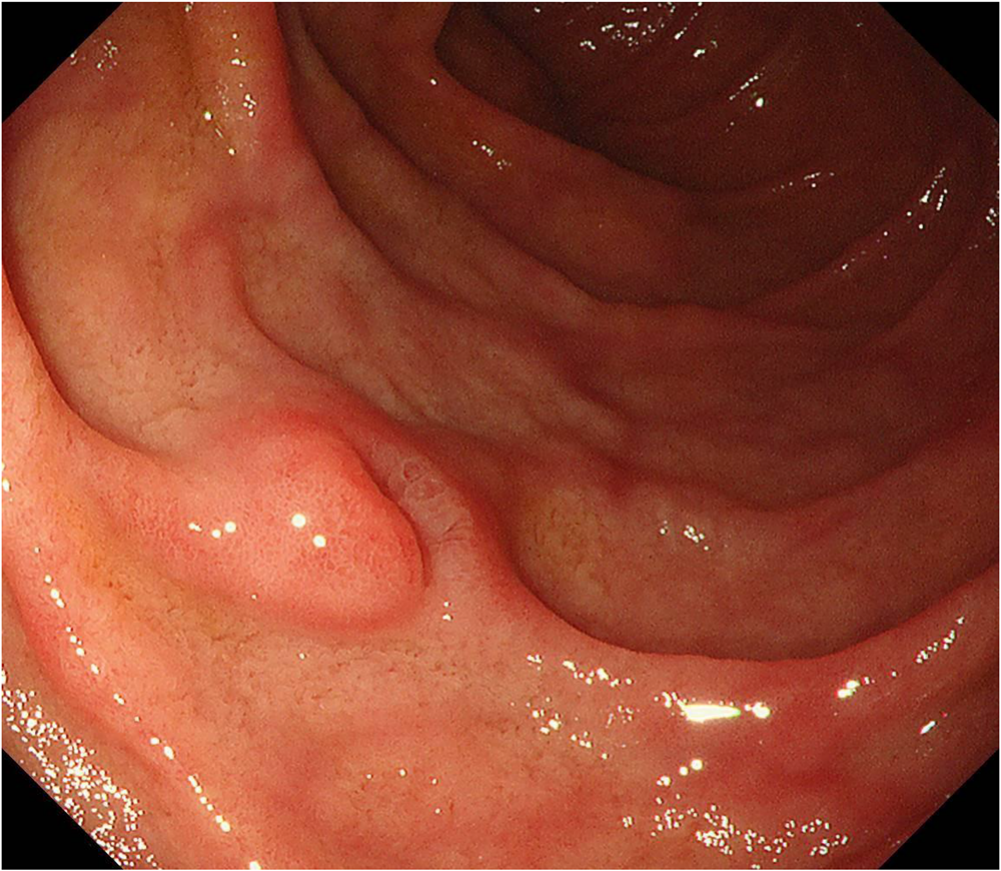
Endoscopic image of superficial non‐ampullary duodenal epithelial tumor. The tumor is present only in the depression of the lesion, and the morphology is 0‐IIc. This lesion pathologically extends into the submucosal layer via Brunner's glands.

The relationship between BG involvement patterns and clinicopathological features of the lesions is shown in Tables 2 and 3. Pattern 4 was observed in eight lesions (7%; 95% confidence interval [CI], 3.1%‐13.4%). Among them, seven lesions (87.5%) were 10 mm in size or larger. Lesions with pattern 4 were significantly correlated with 0‐IIc morphology (odds ratio: 9.60, *p* = 0.004), histological category 4 or 5 according to the Vienna Classification (odds ratio: 5.13, *p* = 0.033), and gastric phenotype (odds ratio: 10.0, *p* = 0.006). BG involvement pattern 4 was observed in three lesions (3.6%) classified as histological category 3. Table 4 shows the characteristics of lesions with BG involvement pattern 4. Six lesions were in the second portion of the duodenum, and two were in the bulb. The lesion size ranged from 8 to 28 mm, with three lesions measuring 20 mm or more and only one lesion measuring less than 10 mm (Figure 3). Histologically, three lesions were category 3, and three lesions had an intestinal mucinous phenotype. The presence of either a lesion size 10 mm or greater or 0‐IIc morphology demonstrated a sensitivity of 100% (8/8), a specificity of 34.9% (37/106), and a false negative rate of 0% (0/8) for predicting BG involvement pattern 4.

**TABLE 2:** The relationship between Brunner's gland (BG) involvement patterns and the clinicopathological features.

	Pattern 1–3 (*n* = 106)	Pattern 4 (*n* = 8)	*p*‐Value	Pattern 1 or 2 (*n* = 54)	Pattern 3 or 4 (*n* = 60)	*p*‐Value
**Lesion size< 10 mm, *n* (%)**						
< 10mm	37 (34.9)	1 (12.5)	0.265	29 (53.7)	9 (15.0)	<0.001
≥ 10mm	69(65.1)	7(87.5)		25 (46.3)	51 (85.0)	
**Morphology, *n* (%)**						
0‐I	13 (12.3)	2 (25.0)	0.004	7 (13.0)	8 (13.3)	0.706
0‐IIa	82 (77.4)	2 (25.0)		38 (70.4)	46 (76.7)	
0‐IIc	10 (9.4)	4 (50.0)		8 (14.8)	6 (10.0)	
SMT‐like	1 (0.9)	0 (0)		1 (1.9)	0 (0)	
**Location, *n* (%)**						
1st	15 (14.2)	2 (25.0)	0.837	5 (9.3)	12 (20.0)	0.143
2nd (pre‐ampulla)	40 (37.7)	3 (37.5)		18 (33.3)	25 (41.7)	
2nd (post‐ampulla)	47 (44.3)	3 (37.5)		28 (51.9)	22 (36.7)	
3rd	4 (3.8)	0 (0)		3 (5.6)	1 (1.7)	
**Histological type, *n* (%)**						
Category 3, low‐grade dysplasia	80 (75.5)	3 (37.5)	0.033	44 (81.5)	39 (65.0)	0.059
Category 4 or 5 high‐grade dysplasia, mucosal carcinoma, or submucosal carcinoma	26 (24.5)	5 (62.5)		10 (18.5)	21 (35.0)	
**Mucinous phenotype, *n* (%)**						
gastric	6 (5.7)	3 (37.5)	0.008	4 (7.4)	5 (8.3)	0.034
intestinal	83 (78.3)	3 (37.5)		46 (85.2)	40 (66.7)	
mixed	17 (16.0)	2 (25.0)		4 (7.4)	15 (25.0)	

Abbreviations: BG, Brunner's gland. SMT, submucosal tumor.

**TABLE 3:** Predictive factors of involvement of superficial non‐ampullary duodenal epithelial tumors (SNADETs) into the submucosa via Brunner's glands (BGs).

	Submucosal BG involvement (pattern 4)			
	negative (*n* = 106)	positive (*n* = 8)	OR (95% confidence interval)	*p*‐Value
**Lesion size, *n***				
≥ 10mm	69	7	3.75 (0.45–31.7)	0.224
< 10mm	37	1	reference	
**Morphology, *n***				
0‐IIc	10	4	9.60 (2.08–44.4)	0.004
others	96	4	reference	
**Location, *n***				
pre‐ampulla	55	5	1.55 (0.35–6.80)	0.565
post‐ampulla	51	3	reference	
**Histological type, *n***				
category 4,5 high‐grade dysplasia, mucosal carcinoma, or submucosal carcinoma	26	5	5.13 (1.15–22.9)	0.033
category 3, low‐grade dysplasia	80	3	reference	
**Mucinous phenotype, *n***				
gastric	6	3	10.0 (1.92–52.1)	0.006
others	100	5	reference	

Abbreviation: BG, Brunner's gland.

**TABLE 4:** Lesion showing submucosal Brunner's gland (BG) involvement (pattern 4).

Case No.	Age	Sex	Location	Size, mm	Morphology	Resection methods	Histological type according to VC	Mucinous phenotype	pHM	pVM	Non‐neoplastic BGs in the vertical margin	Ratio of BG/SNADET	BG hyperplasia	Follow‐up period (month)	Recurrence
**1**	55	Male	1st	28	0‐IIa	ESD/LECS	category 4	gastric	pHM0	pVM0	negative	>76%	positive	49	No
**2**	53	Male	2nd (post‐ampulla)	25	0‐Is	Surgery	category 3	intestinal	pHM0	pVM0	negative	>76%	positive	−	−
**3**	84	Male	2nd (pre‐ampulla)	20	0‐IIc	Surgery	category 4	gastric	pHM0	pVM0	negative	>76%	positive	−	−
**4**	78	Female	2nd (post‐ampulla)	15	0‐IIa	EMR (conventional)	category 3	intestinal	pHM0	pVM0	negative	51%–75%	positive	93	No
**5**	82	Male	1st	10	0‐Is	EMR (conventional)	category 4	gastric	pHM0	pVM0	negative	>76%	positive	32	No
**6**	43	Female	2nd (post‐ampulla)	10	0‐IIc	EMR (conventional)	category 4	mixed	pHMX	pVM0	negative	51%–75%	positive	6	Yes
**7**	72	Male	2nd (pre‐ampulla)	8	0‐IIc	EMR (conventional)	category 4	gastric	pHM0	pVM0	negative	26%–50%	positive	8	No
**8**	57	Male	2nd (pre‐ampulla)	12	0‐IIc	ESD/LECS	category 3	intestinal	pHM0	pVM0	negative	51%–75%	positive	47	No

Abbreviations: BG, Brunner's gland; EMR, endoscopic mucosal resection; ESD, endoscopic submucosal dissection; LECS, laparoscopic endoscopic cooperative surgery; SNADET, superficial non‐ampullary duodenal epithelial tumor; VC, Vienna classification.

## 4 Discussion

In this study, we performed a detailed histopathological evaluation of the relationship between SNADETs and BGs using specimens obtained via endoscopic or surgical resection. We found that BGs were identified in 92% of SNADETs, and 52.6% of the SNADETs had a direct connection with BGs. Notably, 7% of the lesions extended to the submucosal layer via BGs, not infiltrating beyond the basement membrane of BGs into the stroma. This finding was observed in patients with low‐grade dysplasia 10 mm or larger, high‐grade dysplasia, or carcinomas. BGs were also exposed at the vertical resection margin in 15.1% of endoscopic resection specimens.

The association of BGs and SNADETs is worth noting in the era where endoscopic treatment is the primary treatment strategy for SNADETs. While CSP, EMR, UEMR, ESD, and LECS are increasingly used as endoscopic treatments for SNADETs worldwide [4, 17], a definitive strategy based on tumor biology has yet to be established. To our knowledge, our study is the first to histopathologically evaluate the patterns of SNADETs involving BGs, demonstrating that tumors may extend along BGs into the submucosa. This is similar to the relationship between esophageal squamous cell carcinoma and esophageal glands; even superficial neoplasms may progress within the esophageal glands [18]. Our findings suggest that the vertical resectability may deserve consideration, particularly in lesions with risk factors for BG involvement; however, further prospective outcome data are needed to determine its clinical significance.

CSP, although widely used as an endoscopic treatment for small colorectal polyps, has been shown to have limitations in resection depth [19, 20]. It has been reported that the muscularis mucosae remains in 63% of wounds after CSP for colorectal lesions [21]. A recent study showed that the complete resection rate of muscularis mucosae with CSP in the duodenum was only 26.9%, significantly lower than that of UEMR [22]. In a randomized clinical trial, UEMR had significantly deeper and more frequent submucosal resections than CSP, highlighting its superiority in vertical resectability [23]. In the current study, lesions with submucosal BG involvement (pattern 4) included an 8‐mm lesion. It suggests that even small tumors 10 mm or smaller can extend along the BGs beyond the muscularis mucosae. Although CSP is generally performed for small lesions, its long‐term outcomes, such as the recurrence rate of duodenal tumors, remain unclear. One report indicated a 2.7% recurrence rate after CSP for lesions ≤7 mm or small during a longer than 12‐month follow‐up, but the sample size was limited [24]. Given that BG involvement occasionally extends beyond the muscularis mucosae, these findings raise the possibility that resection depth could become relevant in a subset of lesions, particularly when CSP is considered. Nevertheless, current data regarding long‐term clinical outcomes after CSP remain limited, and the clinical implications of our pathological findings should be interpreted with caution.

Submucosal BG involvement was associated with depressed (0‐IIc) morphology, higher histological grade, and gastric mucinous phenotype. Although BGs are generally more dominant proximal to the ampulla, submucosal BG involvement has also been observed in distal lesions, with no obvious association with tumor location. In addition, no submucosal involvement was observed in patients with low‐grade dysplasia smaller than 10 mm. Endoscopic images can predict histological grade and mucin type, but accurate diagnosis is not straightforward [25, 26]. The tumor volume involving BG is small, and it would be difficult to detect even with endoscopic ultrasound. Therefore, we considered lesion size (≥10 mm) and morphology (0‐IIc) as practical predictive factors for submucosal involvement. When we examined lesions meeting either criterion of being ≥10 mm in size or having a 0‐IIc morphology as a predictive factor for submucosal involvement, the sensitivity was 100%, and the false‐negative rate was 0%. This simple combined index of lesion size and morphology may allow the preoperative identification of submucosal involvement.

Our study has several limitations. First, this was a single‐center retrospective analysis with a limited number of patients. Second, specimens resected with CSP were excluded because of a lack of submucosal tissue, and BG evaluation was missing in such cases. However, only two lesions were resected with CSP during the study period, and EMR was performed for most small lesions. Third, specimens surgically resected were sectioned at wider intervals than those endoscopically resected, potentially underestimating the BG involvement. Fourth, our cohort included a few lesions 20 mm or larger, which might need further investigation. Fifth, endoscopic findings that may indicate mucinous phenotype or histological grade have not been examined.

In conclusion, SNADETs can extend into the submucosa via BGs. Lesions with potential submucosal involvement may be suggested by a lesion fulfilling either a size of 10 mm or larger or a 0‐IIc morphology. These pathological findings suggest that vertical resectability may be a relevant consideration in the management of SNADETs; however, further studies are needed to clarify how these histological features translate into clinical outcomes and optimal treatment selection.

## Author Contributions

KT, TI, and MI: conception and design. KT, TI, MI, SS, AH, NS, KO, HY, KM, RT, TT, NK, AT, RT, MA, and SL: data acquisition. TI: formal analysis. KT, TI, MI, and YH: methodology. KT and TI: drafting of the article. All authors have approved the final version of the manuscript.

## Conflicts of Interest

The authors declare no conflicts of interest.

## Funding

No financial support was received for this study.

## Ethics Statement

The study protocol was approved by the Institutional Review Board of Osaka Medical and Pharmaceutical University Hospital (Nos. 2020–124 and 2024–084) and was conducted in accordance with the principles of the Declaration of Helsinki.

## Consent

Informed consent was obtained using the opt‐out method detailed on our institute's website.

## Clinical Trial Registration

Not applicable.

## Supporting information




**FIGURE S1** Histopathological image of Brunner's gland (BG) hyperplasia. The BG indicated by the yellow line shows hyperplasia compared to the neighbor normal BG indicated by the blue line.
**FIGURE S2** Histopathological image of superficial non‐ampullary duodenal epithelial tumor (SNADET) with submucosal invasion. The HE‐stained image (a) shows cancer invading the submucosal layer. The MUC6‐immunostained image (b) shows that Brunner's glands stained with MUC6 (red arrows) are adjacent to the tumor but not infiltrated by the tumor.

**TABLE S1** Comparison of endoscopic resection methods.
**TABLE S2** Characteristics of cases with recurrence after endoscopic resection for SNADTEs.

## Data Availability

The data that support the findings of this study are available on request from the corresponding author.
