# Supplementary figures and images for: Clinicopathological Features of Superficial Non‐Ampullary Duodenal Epithelial Tumors Involving Brunner's Glands
Source: DEN Open. 2026 Jan 26;6(1):e70284. doi: 10.1002/deo2.70284 (PMC12834701; doi:10.1002/deo2.70284)

## Slide 1
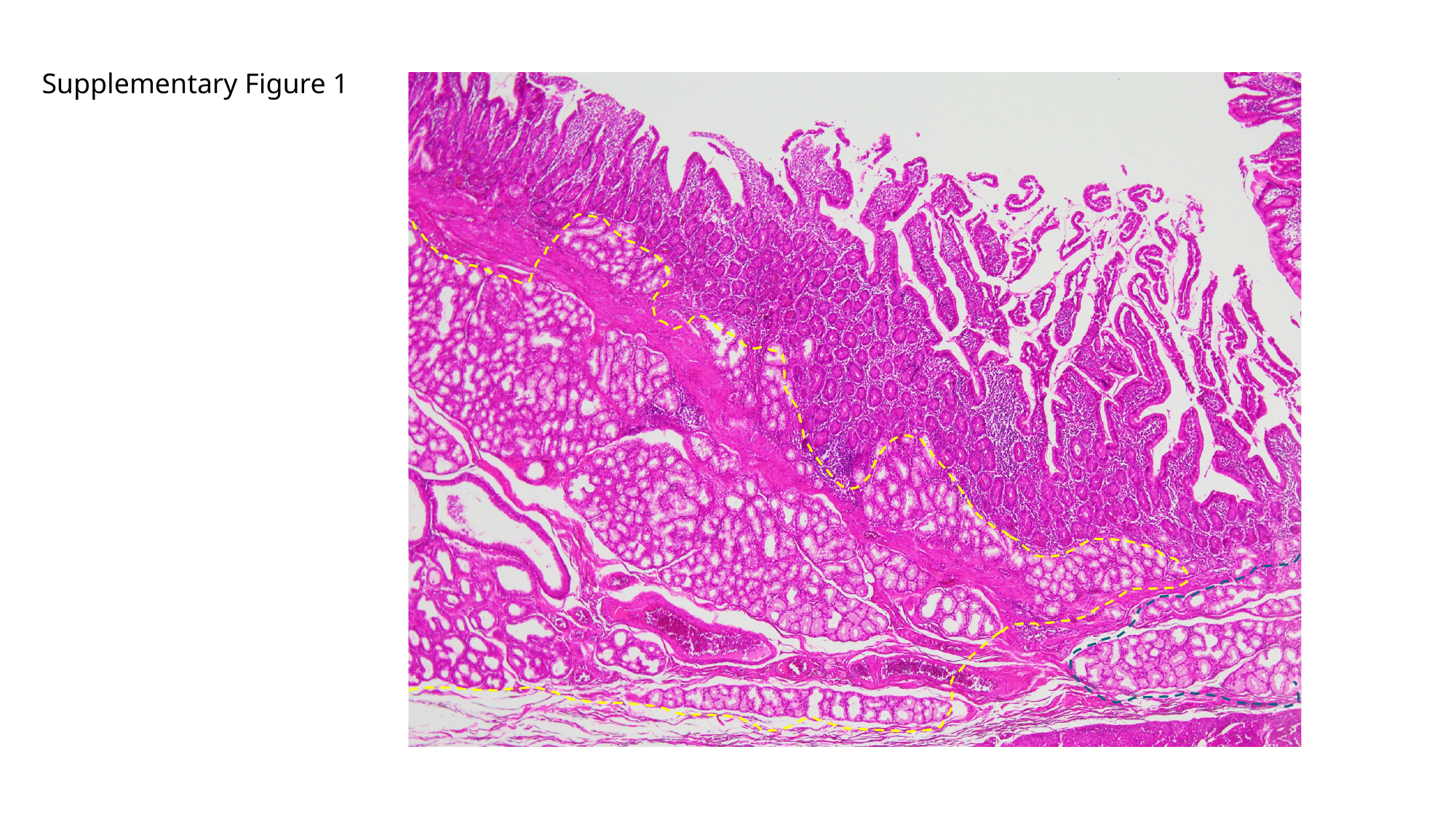

Supplementary Figure 1

## Slide 2
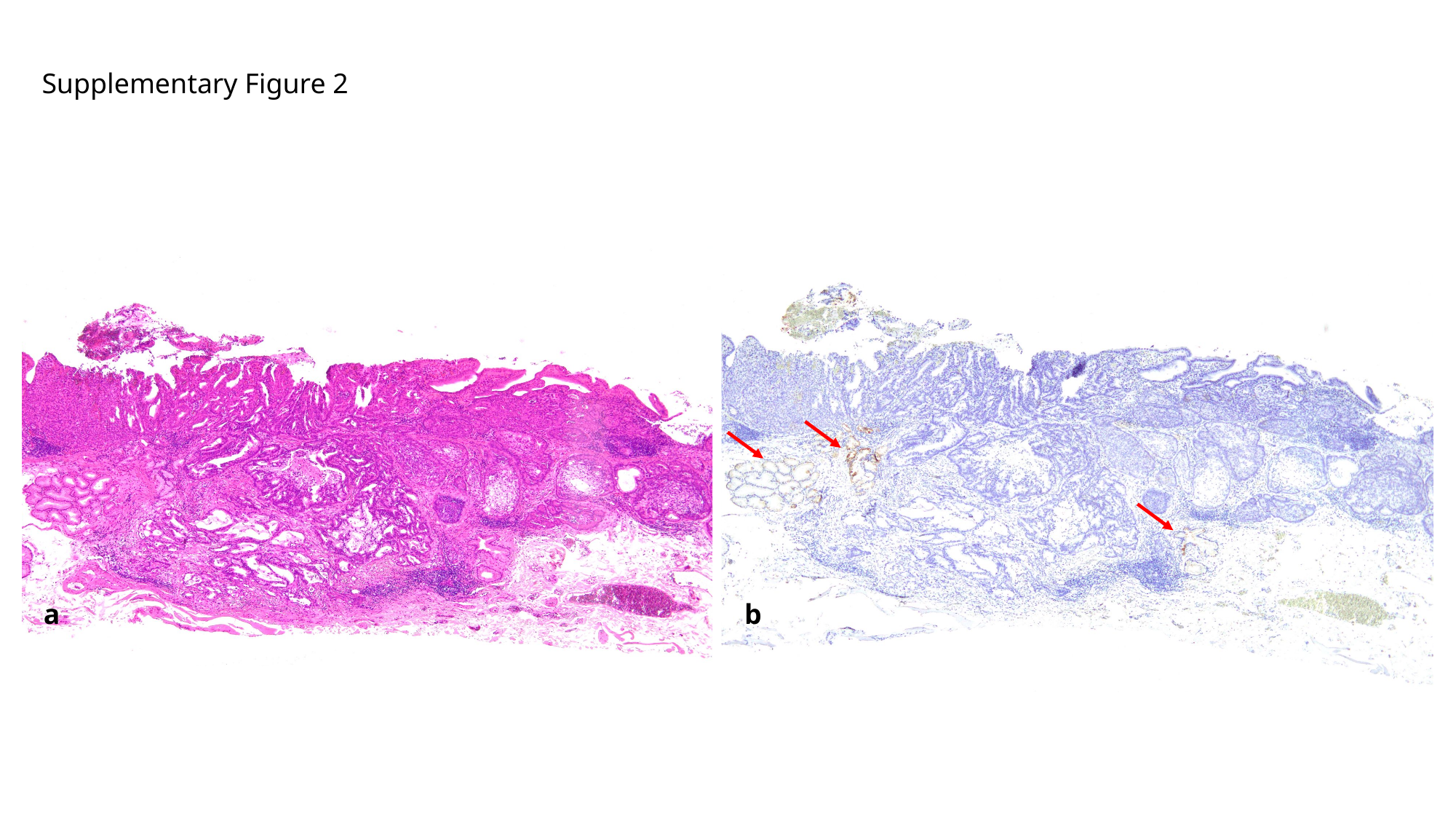

Supplementary Figure 2
a
b

Supplement: Supplementary file 1 — FIGURE S1 Histopathological image of Brunner's gland (BG) hyperplasia. The BG indicated by the yellow line shows hyperplasia compared to the neighbor normal BG indicated by the blue line. FIGURE S2 Histopathological image of superficial non‐ampullary duodenal epithelial tumor (SNADET) with submucosal invasion. The HE‐stained image (a) shows cancer invading the submucosal layer. The MUC6‐immunostained image (b) shows that Brunner's glands stained with MUC6 (red arrows) are adjacent to the tumor but not infiltrated by the tumor. [file DEO2-6-e70284-s002.pptx]
