# Supplementary material for: Clinicopathological Features of Superficial Non‐Ampullary Duodenal Epithelial Tumors Involving Brunner's Glands
Source: DEN Open. 2026 Jan 26;6(1):e70284. doi: 10.1002/deo2.70284 (PMC12834701; doi:10.1002/deo2.70284)
Supplement: Supplementary file 2 — TABLE S1 Comparison of endoscopic resection methods. TABLE S2 Characteristics of cases with recurrence after endoscopic resection for SNADTEs. [file DEO2-6-e70284-s001.docx]

**Supplementary Table 1**. **Comparison of endoscopic resection methods**

|  |  |  | EMR (n=82) | ESD/LECS (n=17) | *P*-value |
| --- | --- | --- | --- | --- | --- |
| Lesion size, mm |  |  |  |  |  |
|  | Mean (SD) |  | 10.9 (5.5) | 22.4 (9.5) | <0.001 |
|  | Medium (range) |  | 10 (3-25) | 15 (7-30) | <0.001 |
| En bloc resection | n (%) |  | 69 (84.1) | 16 (94.1) | 0.682 |
| Resection margin, n (%) |  |  |  |  |  |
|  | Horizontal |  |  |  | 0.600 |
|  |  | pHM0 | 75 (91.4) | 17 (100) |  |
|  |  | pHM1 or X | 7 (8.5) | 0 (0) |  |
|  | Vertical |  |  |  | 1 |
|  |  | pVM0 | 82 (100) | 17 (100) |  |
|  |  | pVM1 or X | 0 (0) | 0 (0) |  |
| Non-neoplastic BGs in the vertical margin, n (%) |  |  |  |  |  |
|  | negative |  | 68 (82.9) | 16 (94.1) | 0.457 |
|  | positive |  | 14 (17.1) | 1 (5.9) |  |
|  |  |  |  |  |  |
| Follow-up period ≥ 1 month |  |  | n=67 | n=13 |  |
|  | Follow-up period, median (range), month |  | 25 (1-123) | 40 (2-107) | 0.399 |
|  | Recurrence during follow-up, n (%) |  | 4 (6.0) | 0 (0) | 1 |

BG, Brunner’s gland; EMR, endoscopic mucosal resection; ESD, endoscopic submucosal dissection; LECS, laparoscopic endoscopic cooperative surgery; SD, standard deviation; SMT, submucosal tumor.

| Supplementary Table 2. Characteristics of cases with recurrence after endoscopic resection for SNADTEs | | | | | | | | | | | | | | |
| --- | --- | --- | --- | --- | --- | --- | --- | --- | --- | --- | --- | --- | --- | --- |
| Age | Sex | Location | Size, mm | Morphology | Resection methods | En bloc resection | pHM | pVM | Histological type according to VC | BGs involvement patterns | Mucinous phenotype | Non-neoplastic BGs in the vertical margin | the ratio of BG/SNADET | BG hyperplasia |
| 43 | Female | 2nd (post-ampulla) | 10 | 0-IIc | EMR (conventional) | Yes | pHMX | pVM0 | category 4 | pattern 4 | mixed | negative | 51-75% | positive |
| 74 | Male | 3rd | 18 | 0-Is | EMR (underwater) | Yes | pHM0 | pVM0 | category 3 | pattern 3 | intestinal | negative | 1-25% | negative |
| 85 | Female | 2nd (pre-ampulla) | 25 | 0-Is | EMR (underwater) | No | pHM1 | pVM0 | category 3 | pattern 3 | intestinal | positive | 51-75% | positive |
| 72 | Male | 2nd (pre-ampulla) | 8 | 0-IIa | EMR (conventional) | Yes | pHMX | pVM0 | category 3 | pattern 2 | intestinal | positive | >76% | negative |

BG, Brunner’s gland; EMR, endoscopic mucosal resection; SNADET, superficial non-ampullary duodenal epithelial tumor; VC, Vienna classification.
